# Supplementary material for: Predicting visual function by interpreting a neuronal wiring diagram
Source: Nature. 2024 Oct 2;634(8032):113–23. doi: 10.1038/s41586-024-07953-5 (PMC11446822; doi:10.1038/s41586-024-07953-5)
Supplement: Supplementary file 5 — CRF and ERF predictions for individual Dm3 cells. The CRF is predicted for each target cell by mapping the top five monosynaptic pathways from hexel source types to the target Dm3 type. The ERF is predicted using disynaptic pathways that pass through the top ten intermediary types (ranking from Extended Data Fig. 7). As a set of disynaptic pathways passes from multiple hexel source types through each intermediary to the target, for brevity only one representative starting from a single hexel type is shown, the pathway in the set with maximal strength (red line, Extended Data Fig. 7). The centre of the Tm1–Dm3 CRF (Methods) is indicated by the cyan star, to aid comparison of locations across maps. Next to each map are the maximum and sum over all hexels, in units of 0.01%. In the last panel, all ellipse approximations are shown together for comparison, scaled up by ×3 relative to maps. The dashed ellipse approximates the Tm1–Dm3 CRF, and scale bars indicate one lattice constant and p and q axes. Each file name contains the ID of the target cell in v783. [file 41586_2024_7953_MOESM5_ESM.zip › DataS3/Dm3q/720575940614415411.pdf]

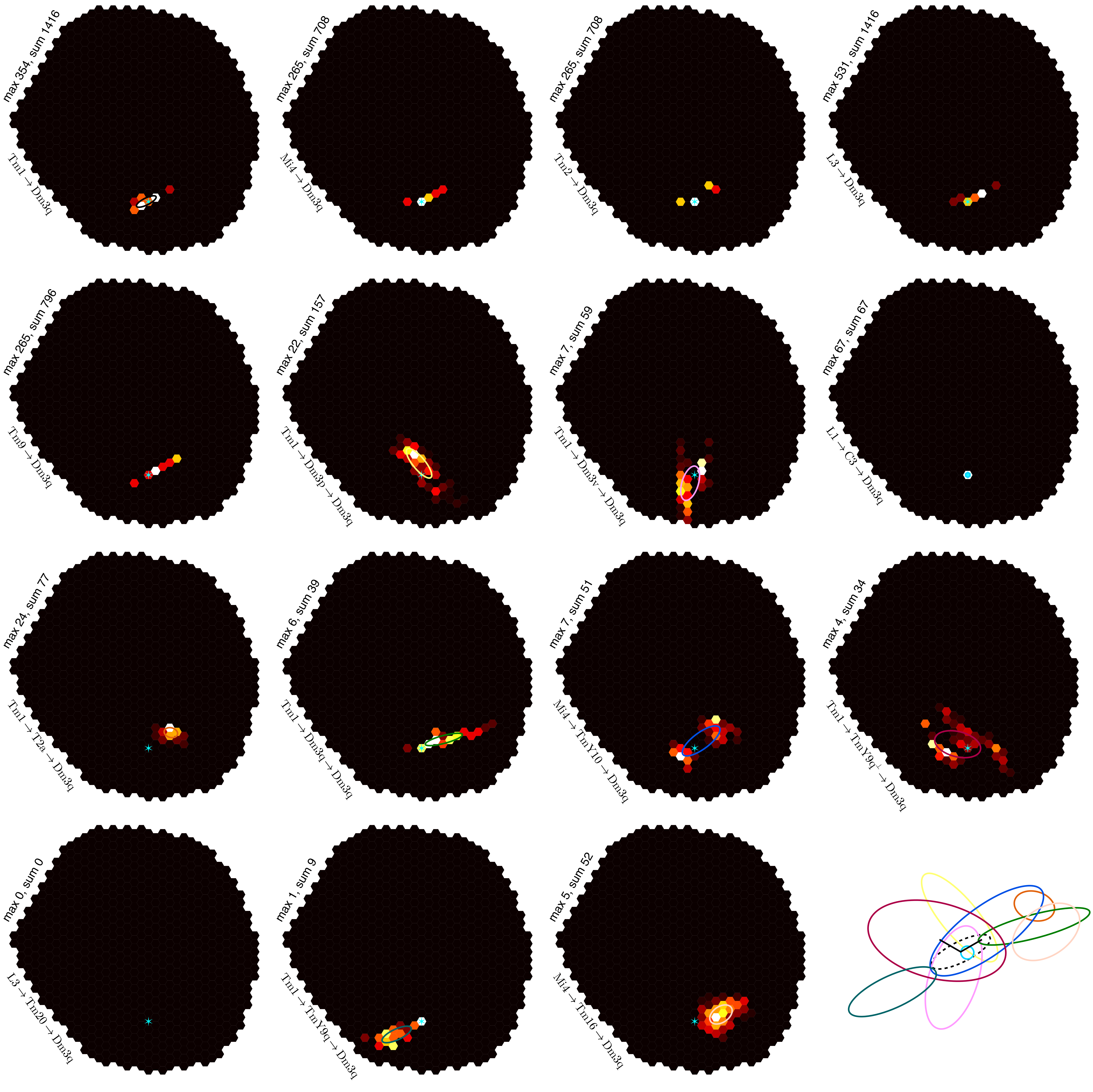

max 354, sum 1416
Tm1 → Dm3q
max 265, sum 708
Mi4 → Dm3q
max 265, sum 708
Tm2 → Dm3q
max 531, sum 1416
L3 → Dm3q
max 265, sum 796
Tm9 → Dm3q
max 22, sum 157
Tm1 → Dm3p → Dm3q
max 7, sum 59
Tm1 → Dm3v → Dm3q
max 67, sum 67
L1 → C3 → Dm3q
max 24, sum 77
Tm1 → T2a → Dm3q
max 6, sum 39
Tm1 → Dm3q → Dm3q
max 7, sum 51
Mi4 → TmY10 → Dm3q
max 4, sum 34
Tm1 → TmY9q⊥ → Dm3q
max 0, sum 0
L3 → Tm20 → Dm3q
max 1, sum 9
Tm1 → TmY9q → Dm3q
max 5, sum 52
Mi4 → Tm16 → Dm3q
